# Supplementary material for: Structural Basis for the Catalysis and Substrate Specificity of a LarA Racemase with a Broad Substrate Spectrum
Source: ACS Catal. 2025 Feb 3;15(4):2857–66. doi: 10.1021/acscatal.4c07804 (PMC11851776; doi:10.1021/acscatal.4c07804)
Supplement: Supplementary file 1 — cs4c07804_si_001.pdf [file cs4c07804_si_001.pdf]

Supplementary information

Structural Basis for Catalysis and Substrate Specificity of a LarA Racemase with a Broad Substrate Spectrum

Santhosh Gatreddi[1,2], Julian Urdiain-Arraiza[3], Benoit Desguin[3,*], Robert P. Hausinger[1,2,*], Jian Hu[2,4,*]

[1]Department of Microbiology, Genetics, and Immunology, Michigan State University, MI 48824

[2]Department of Biochemistry and Molecular Biology, Michigan State University, MI 48824

[3]Louvain Institute of Biomolecular Science and Technology (LIBST), Université catholique de Louvain, B-1348 Louvain-La-Neuve, Belgium

[4]Department of Chemistry, Michigan State University, MI 48824

*Corresponding authors: Benoit Desguin (benoit.desguin@uclouvain.be); Robert P. Hausinger (hausinge@msu.edu); Jian Hu (hujian1@msu.edu)

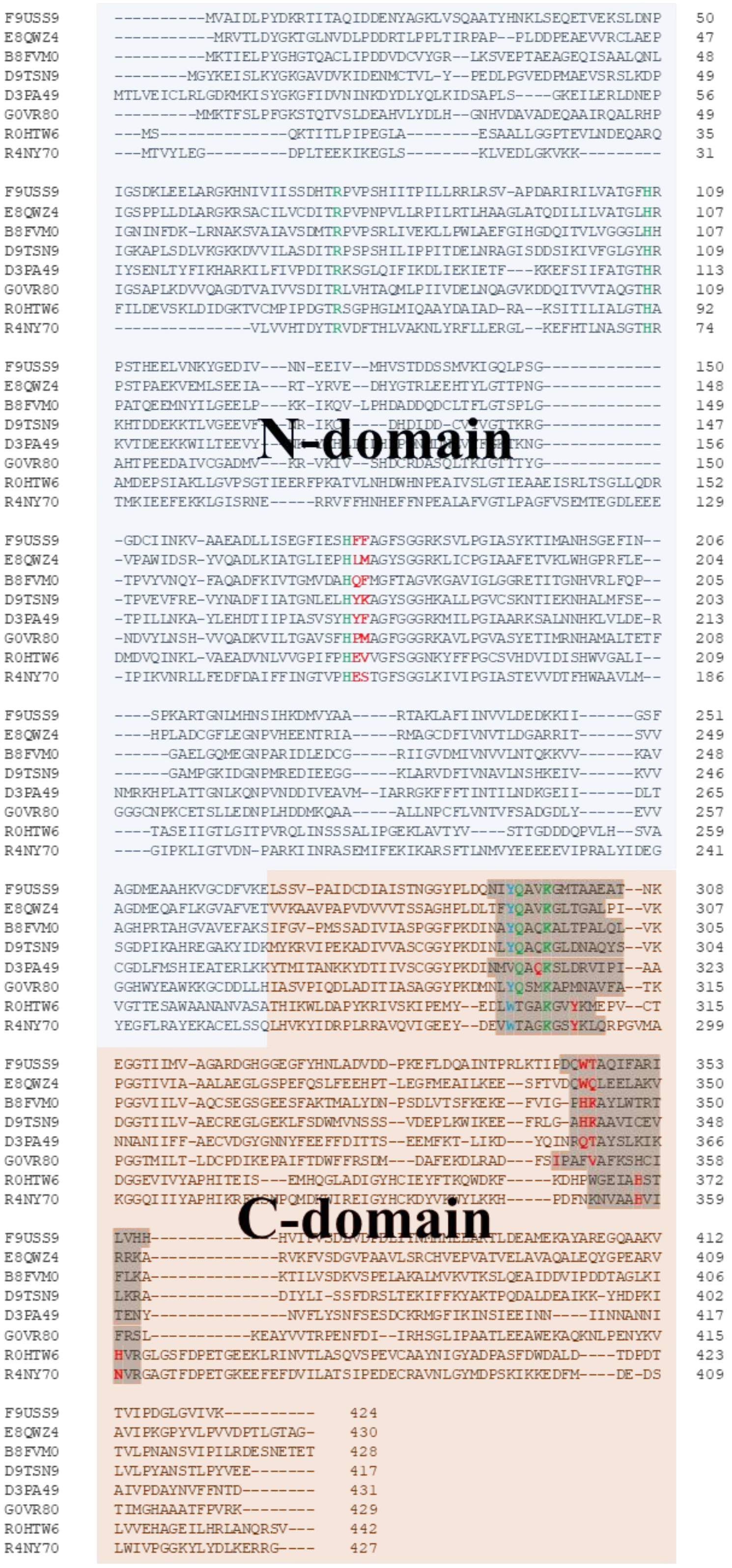

Figure S1. Sequence alignment of LarA enzymes with known substrates. The Uniprot IDs and the corresponding LarA enzymes are listed below. F9USS9 – LarA$_{Lp}$ (group 1); E8QWZ – LarA$_{Ip}$

(group 2); B8FVM0 – Mar (group 5); D9TSN9 – Mar2 (group 6); D3PA49 – Hgr (group 7); G0VR80 – Plr (group 10); R0HTW6 – GntE1 (group 19); R4NY70 – GntE2 (group 20). The colored residues are shown for $LarA_{lp}$ or predicted (for all others) to directly interact with D-α-hydroxyacid substrates: red – Cα substituent; green – carboxylic acid group; blue – α-hydroxyl group. The shaded sequences indicate the two α-helices that bind substrates from the C-terminal domain. The N- and C-terminal domains are shown in the light blue and brown boxes, respectively.

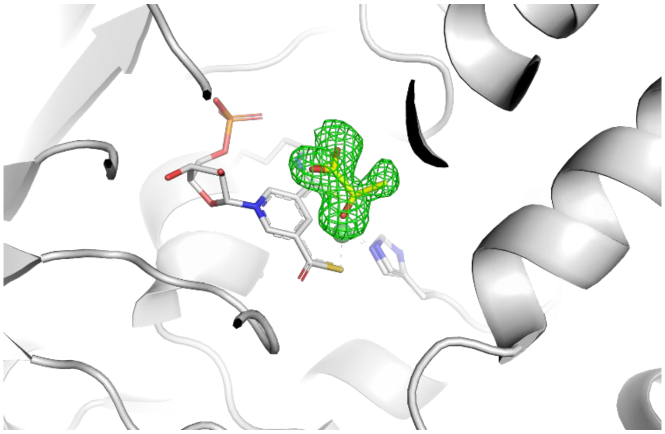

Figure S2. Fo-Fc omit map (green meshes, σ=3) of D-lactate (stick mode in yellow) in $LarA_{lp}$ as purified (Chain A). D-lactate, the NPN cofactor, Lys183, and His199 are shown in stick mode.

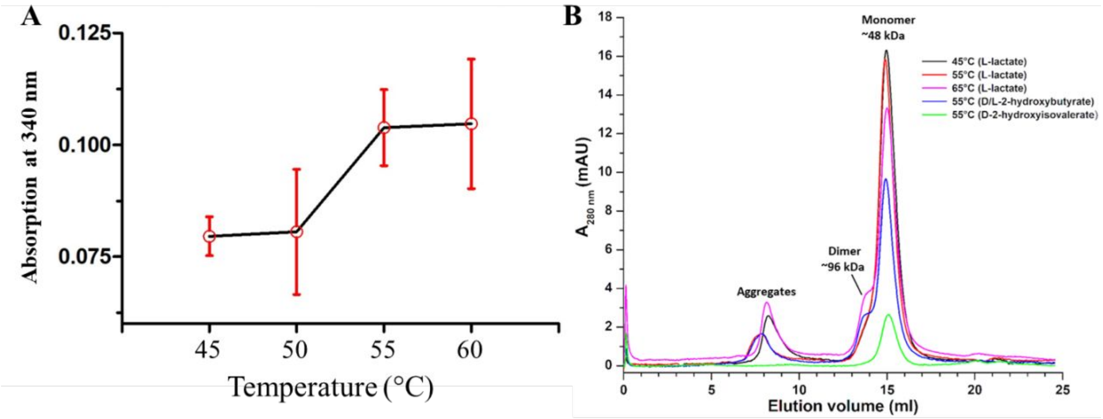

Figure S3. Heat stability test of LarA_{lp}. (**A**) Temperature dependence of LarA_{lp} activity. L-lactate racemase activity was measured at the indicated temperatures. The error bars indicate standard deviations (n=3). (**B**) Heat stability test of LarA_{lp}. Monomeric LarA_{lp} as purified (4 μM) was heated at the indicated temperatures in 20 mM Tris-HCl (pH 7.5) and 125 mM NaCl for 30 min in the presence of 3-5 mM substrates and then cooled rapidly to 4 °C. The stability of the treated samples was evaluated by size-exclusion chromatography.

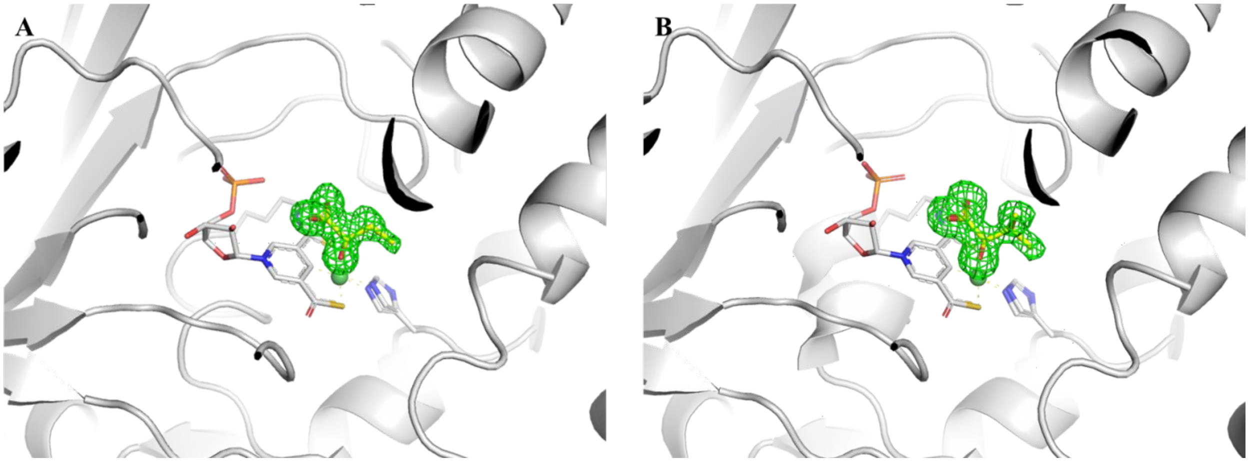

Figure S4. Fo-Fc omit maps (green mesh, σ=3) of LarA$_{lp}$ after ligand exchange with D-2HB (left) and D-2HIV (right). Substrates (yellow), the NPN cofactor, Lys183, and His199 are shown in stick mode.

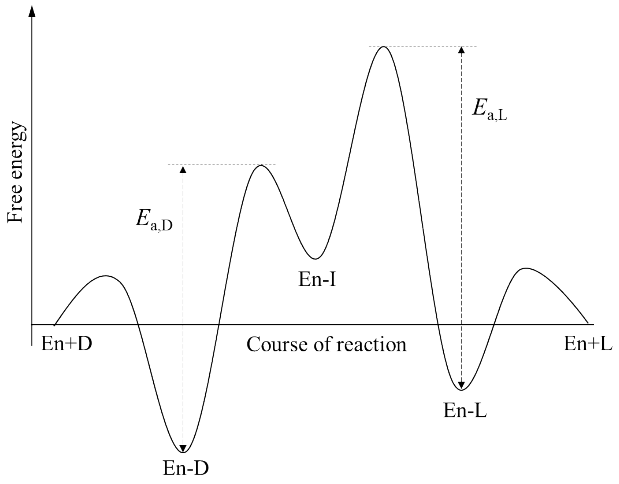

Figure S5. Proposed free energy profile of the racemization reaction catalyzed by LarA$_{lp}$. En: enzyme. D: D-enantiomer. L: L-enantiomer. En-D/L: enzyme-substrate complex. En-I: enzyme-intermediate complex. $E_{a,D/L}$: activation energy of the racemization reaction using D- or L-enantiomer as substrate.

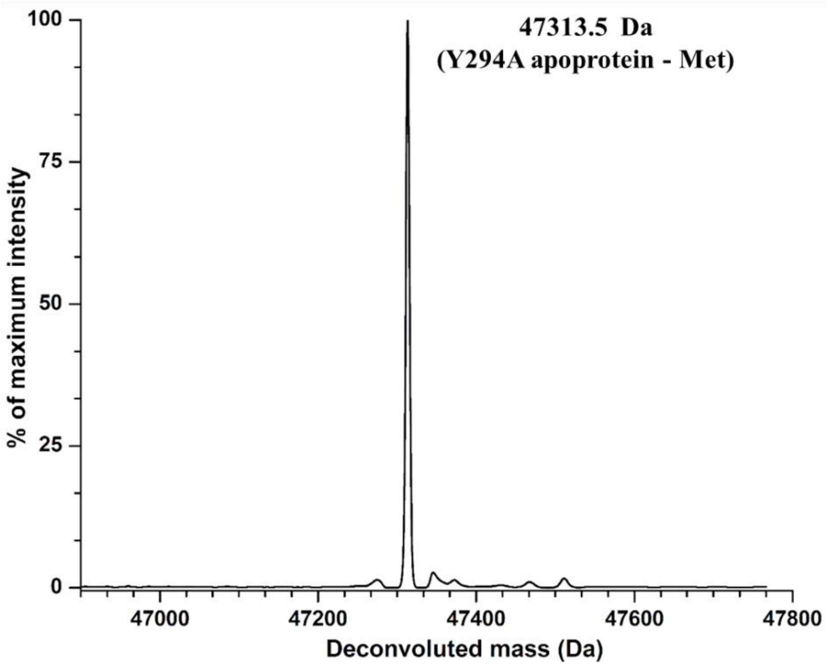

Figure S6. ESI-MS of the Y294A variant as purified from *L. lactis*.

Table S1. k_{cat}/K_M values of LarA$_{lp}$ for α-hydroxyacids.

Reactions/Substrates	Relative k_{cat}/K_M (%)	k_{cat}/K_M (M^{-1} s^{-1})
D- ↔ L-lactate	100	(31 ± 9) x 10^3
D- ↔ L-2-hydroxybutyrate	95 ± 3	(30 ± 8) x 10^3
D- ↔ L-glycerate	26 ± 5	(82 ± 27) x 10^2
D- ↔ L-2,4-dihydroxybutyrate	16 ± 3	(51 ± 16) x 10^2
D- ↔ L-2-hydroxyvalerate	9.1 ± 0.9	(28 ± 8) x 10^2
D- ↔ L-2-hydroxyisovalerate	6.2 ± 1.5	(19 ± 7) x 10^2
D- ↔ L-2-hydroxycaproate	5.7 ± 1.1	(18 ± 6) x 10^2
4-deoxy-L-threonate ↔ 4-deoxy-L-erythronate	4.8 ± 1.2	(15 ± 5) x 10^2
D- ↔ L-2-hydroxyisocaproate	3.4 ± 0.4	(11 ± 3) x 10^2
4-deoxy- D-threonate ↔ 4-deoxy-D-erythronate	2.8 ± 0.5	(89 ± 28) x 10^1
D-threonate ↔ L-erythronate	1.8 ± 0.4	(56 ± 20) x 10^1
D-threonate ↔ D-erythronate	1.3 ± 0.3	(42 ± 14) x 10^1
D- ↔ L-3-phenyllactate	0.85 ± 0.09	(27 ± 8) x 10^1
D- ↔ L-2-hydroxy-4-phenylbutyrate	0.39 ± 0.08	(13 ± 4) x 10^1

Table S2. Crystallographic statistics.

Data collection	**$LarA_{lp}$ as purified (D-lactate)**	**D-2-hydroxybutyrate**	**D-2-hydroxyisovalerate**
Beamline	LS-CAT 21-ID-D	NSLSII 17-ID-2 FMX	NSLSII 17-ID-2 FMX
Wavelength (Å)	1.127231	0.97934	0.97934
Space group	$P2_1$	$P2_12_12_1$	$P2_1$
Unit cell a, b, c (Å);	79.42, 45.51, 119.14	46.72, 79.61, 104.78	79.02 45.25 118.22
α, β, γ (°)	90.00, 91.20, 90.00	90.00, 90.00, 90.00	90.00 91.07 90.00
[a]Resolution (Å)	33.07 – 1.74 (1.80-1.74)	29.11 – 1.38 (1.41-1.38)	29.71 – 1.65 (1.68-1.65)
[a]Redundancy	2.5 (2.0)	12.8 (7.8)	6.9 (6.9)
[a]Completeness (%)	96.2 (94.3)	99.7 (95.7)	99.1 (93.4)
[a]$I/\sigma I$	10.3 (1.52)	18.6 (2.9)	10.9 (2.6)
[a,b]R_{merge}	0.112 (0.567)	0.095 (0.763)	0.118 (0.716)
[a,c]R_{pim}	0.083 (0.469)	0.027 (0.288)	0.048 (0.291)
[d]$CC_{1/2}$ of the highest resolution shell	0.610	0.805	0.762
**Refinement**			
Unique reflections	84,457	80,218	100,330
Number of atoms	6950	3850	7165
Protein atoms	6339	3280	6440
H_2O molecules	516	528	635
Phosphate	0	2	0
EDO	3	0	0
PEG	3	0	2
PGE	0	0	1
Substrate (D-lactate/D-2-hydroxybutyrate/D-2-isovalerate)	2	1	2
Ni	2	1	2
4EY	2	1	2
[e]R_{work}/R_{free}	0.175/0.212	0.152/0.166	0.167/0.191
B-factors (Å^2)	18.6	13.7	17.3
Protein atoms	17.9	12.2	16.5
H_2O molecules	26.4	23.4	25.9
Phosphate	-	21.4	-
EDO	34.0	-	-
PEG	36.8	-	36.1
PGE	-	-	32.3
Substrate (D-lactate/D-2-hydroxybutyrate/D-2-isovalerate)	15.3	12.5	12.7
Ni atoms	12.3	7.4	11.7
4EY: P2TMN	11.1	7.6	10.8
R.m.s. deviation in bond lengths (Å)	0.007	0.006	0.007
R.m.s. deviation in bond angles (°)	0.982	0.990	1.01
Ramachandran plot (%) favored	98.5	98.4	98.3
Ramachandran plot (%) allowed	1.5	1.6	1.7
Ramachandran plot (%) outliers	0	0	0
Rotamer (%) outliers	0	0.56	0.3
PDB ID	9EIA	9EID	9EIF

[a]Highest resolution shell is shown in parentheses.
[b]$R_{merge} = \sum_{hkl} \sum_j |I_j(hkl) - \langle I(hkl) \rangle| / \sum_{hkl} \sum_j I_j(hkl)$, where *I* is the intensity of reflection.
[c]$R_{pim} = \sum_{hkl} [1/(N-1)]^{1/2} \sum_j |I_j(hkl) - \langle I(hkl) \rangle| / \sum_{hkl} \sum_j I_j(hkl)$, where N is the redundancy of the dataset.
[d]$CC_{1/2}$ is the correlation coefficient of the half datasets.

[e]$R_{work} = \sum_{hkl} \mid |F_{obs}| - |F_{calc}| \mid / \sum_{hkl} |F_{obs}|$, where F_{obs} and F_{calc} is the observed and the calculated structure factor, respectively. R_{free} is the cross-validation R factor for the test set of reflections (5% of the total) omitted in model refinement.

Table S3. Strains, plasmids, and primers used in this study.

Strain, plasmid or primer	Characteristic(s) or sequence	Note
**Strains**		
L. lactis (NZ3900)	MG1363 derivative	
**Plasmids**		
pGIR210-LarAH31	Chl^r. Production of $LarA_{lp}$ fused with a C-terminal Strep-tag	this study
pGIR210-Y294A	Chl^r. Production of the Y294A variant fused with a C-terminal Strep-tag	this study
**Primers (5’-3’)**		
LarAH31 Y294A-F	GATCTGACCTTCGCCCAAGCGGTGAAAG	mutagenesis
LarAH31 Y294A-R	CTTTCACCGCTTGGGCGAAGGTCAGATC	mutagenesis
LarAH31-SR	GTTGTAATATTTCTGCTGTGGTTGCC	sequencing
UP_PNZ8048’	ACAATGATTTCGTTCGAAGGAACTAC	sequencing
